# Modification of low nickel biograde stainless steel with graphene oxide for enhanced corrosion resistance and in vivo biocompatibility

**DOI:** 10.1038/s41598-025-01838-x

**Published:** 2025-05-17

**Authors:** Doaa A. Abu Muslim, Amal S. Shahat, A. B. El Basaty, A. Hassen, A. Abou Elfadl, Ahmed I. Ali, A. Tayel

**Affiliations:** 1 Physics Department, Faculty of Science, Fayoum University, El Fayoum, 63514 Egypt; 2 Basic Science Department, Faculty of Technology and Education, Helwan University, Saraya-El Koba, El Sawah Street, Cairo, 11281 Egypt; 3 General Division of Main Medical Science, Egyptian Drug Authority (EDA), Formally National Organization for Drug Control and Research (NODCAR), Cairo, 12611 Egypt; 4 Nanotechnoloy Centre, Helwan University, Helwan Al Sharqia, Cairo, 11722 Egypt; 5 Department of Mechanical Engineering (Integrated Engineering Program), Kyung Hee University, 1732 Deogyeong-Daero, Yongin, Gyeonggi 17104 Republic of Korea

**Keywords:** Graphene oxide (GO), Low nickel bio-grade stainless steel, Corrosion resistance, Biocompatibility, Albino rats, Hematological parameters, Biological indices, Physiology, Materials science

## Abstract

This study investigates the integration of graphene oxide (GO) into low nickel bio-grade stainless steel (LNBGSS) to enhance its corrosion resistance and assess its biocompatibility. Three concentrations of GO (0.5, 1.0, and 1.5 wt%) were added to the steel matrix using the powder metallurgy method and annealed in a nitrogen environment. X-ray diffraction and field-emission scanning electron microscopy analyses reveal that while the crystal structure of the steel remains largely unchanged, the morphology of the prepared samples exhibits minimal alteration post-GO integration. The average particle sizes (*D*_av_) of the studied samples were calculated. It was found that *D*_av_ slightly changed with the content of GO. Based on the electrochemical analysis, the inhibition efficiency was determined in different ways and it increased markedly with increasing GO content in LNBGSS composites. Subsequently, biocompatibility assessment was conducted through in vivo studies on albino rats. Thirty-six rats were randomly allocated into six groups. The hematological parameters revealed a nonsignificant (*P* > 0.05) difference except for the rats treated with the low-nickel bio-grade stainless steel powder (LNBGSS) (S0), which had the lowest complete blood count in comparison with other groups. In spite, the hematological parameters of all groups were within the normal reference ranges. The biochemical indices also were not significantly (*P* > 0.05) different by assessment of liver enzymes and kidney functions for all examined groups. These findings suggested that the use of GO in modifying low nickel bio-grade stainless steel alloy is biologically safe and recommendable for enhancing this alloy’s properties.

## Introduction

Metals and alloys are widely utilized as biomaterials in various medical and biological applications due to their unique properties, such as strength, ductility, biocompatibility, and corrosion resistance. These materials play a crucial role in the design and fabrication of medical implants, prosthetics, surgical instruments, and biomedical devices, where mechanical integrity and compatibility with biological systems are essential^1^.

The most commonly used metallic biomaterials include stainless steels, cobalt–chromium-based alloys, titanium, and its alloys, as well as tantalum, niobium, and gold^2^. Stainless steels are particularly prominent in the production of orthopedic implants (e.g., bone plates, screws), cardiovascular devices (e.g., stents, heart valves), dental implants, and surgical instruments such as forceps, needle holders, and retractors. Their popularity is due to their excellent properties, including cost-effectiveness, strength, and corrosion resistance. There are several types of stainless steels, including austenitic, martensitic, ferritic, precipitation-hardening, and duplex stainless steels^3^.

One type of stainless steel commonly used in medical applications is AISI 316L. This alloy is favored for its low cost, good mechanical properties, ease of processing, and composition featuring low carbon content and high levels of nickel and chromium^4^. Despite these advantages, the AISI 316L has notable drawbacks. Studies have shown that it is prone to various forms of corrosion in body fluid environments, including crevice, pitting, intergranular, and excitant corrosion. The most significant concern is the release of metal ions from implant devices due to corrosion. Given that AISI 316L contains approximately 12% nickel, addressing these issues is crucial for its continued use and development as a medical stainless steel^5^.

Nickel is recognized as one of the harmful elements in medical stainless steel. Where the nickel ions can act as allergens in the human body, leading to inflammatory reactions such as swelling, skin itching, eczema, and in higher doses or certain forms, nickel is toxic to both humans and animals. Inflammatory and allergic responses to nickel have been observed in patients with orthopedic, dental, and other types of stainless-steel implants. Due to these concerns, the development of new surgical stainless steels and modifications to ASTM medical standards have focused on reducing nickel content while increasing nitrogen levels. This shift aims to maintain the beneficial properties of stainless steel while minimizing the adverse health effects associated with nickel exposure^5–14^.

Biocompatibility is a widely used term in biomaterials science, yet significant uncertainty remains regarding its precise definition and the mechanisms underlying the phenomena that collectively define it. Biomaterials are increasingly applied in diverse and complex fields, including tissue engineering, invasive sensors, drug delivery, gene transfection systems, nanotechnology-based medical applications, and traditional implantable medical devices. This uncertainty regarding the mechanisms and conditions of biocompatibility has become a major obstacle to advancing these technologie^15^. Biocompatibility testing is a fundamental requirement for developing and regulatory approval of orthopedic materials for clinical use. Biomaterials must meet essential biocompatibility standards outlined by the International Standards Organization (ISO 10993)^16^, ensuring they are nontoxic, nonthrombogenic, noncarcinogenic, nonantigenic, and nonmutagenic to elicit an appropriate biological response^17^. Hematological assessment is one of the most important biocompatibility tests, Hematology testing includes the assessment of the complete blood count. Hemocompatibility assessment is a crucial aspect of evaluating the toxicity of graphene oxide (GO)^17,18^.

On the other hand, there have been significant advancements in nanotechnology, which is increasingly preferred in various fields such as electronics, textiles, environmental applications, medicine, and materials science^19^. Nanotechnology plays a critical role in developing stainless steel materials for biomedical applications. Incorporating nanostructured materials in manufacturing stainless steel has led to surgical instruments, medical equipment, and implants with enhanced corrosion resistance, improved surface functionality, and better biocompatibility. These improvements make stainless steel materials safer and more effective for medical use, addressing many of the issues associated with traditional stainless steel, such as nickel-related allergic reactions and corrosion problems. Integrating nanomaterials into stainless steel is paving the way for next generation biomaterials that offer superior performance and safety in medical applications^20,21^.

Graphene oxide (GO) is considered one of the most important nanomaterials and is a prominent member of the 2D carbon allotropes family. GO consists of graphene sheets with various oxygen-containing functional groups, such as carboxyl groups, epoxide, and hydroxyl, attached to the basal plane and edges. It has been widely used in several fields, including energy storage, nanoelectronics devices, and biomedical applications^22–24^. The advantages of GO biocompatibility according to several studies are shown in its ability to facilitate cellular adhesion, proliferation, and differentiation^25,26^. Also, the usage of GO as a supportive material for tissue engineering applications in many types of surgeries is highlighted due to its advantages. In addition, GO has the potential to catalyze the creation of bioengineered tissues, bone regeneration, and wound healing by creating an environment that is favorable for cellular growth^27^. Moreover, GO interactions with immune cells have raised intriguing possibilities for modulating immune responses during surgical interventions^28,29^. This presents the potential to reduce inflammation, enhance tissue integration, and ultimately improve patient recovery and comfort post-surgery^29^.

The potential applications of GO are undeniably exciting, but several challenges must be addressed to ensure safe and effective clinical implementation. The synthesis of graphene oxide materials suitable for surgical use, long-term biocompatibility assessments, and regulatory approvals are among the foremost challenges. Additionally, the development of standardized surgical protocols and techniques for incorporating graphene into existing procedures is essential to ensure seamless integration and optimal outcomes^30,31^. As the frontiers of materials science and surgery intersect, GO emerges as a transformative force poised to reshape the landscape of surgical practices^32^. Its extraordinary mechanical, electrical, and biocompatible properties offer novel solutions to age-old challenges while presenting unprecedented opportunities for innovation^33,34^. In this context, incorporating GO into the bulk composition of stainless steel provides superior long-term efficiency compared to surface coatings^35^. While GO coatings may degrade, crack, or delaminate over time, embedding GO within the steel matrix provides permanent, uniform reinforcement, significantly enhancing corrosion resistance and mechanical durability. Furthermore, GO-reinforced stainless steel ensures consistent biocompatibility, minimizing wear debris and reducing ion leaching, making it a promising material for advanced biomedical applications such as orthopedic implants and surgical tools^36,37^.

To integrate nanomaterials into stainless steel, choosing the right manufacturing method is crucial. Powder metallurgy emerges as a cornerstone, especially for medical applications like orthopedic implants, cardiovascular devices, dental implants, and surgical instruments. This technique offers unmatched advantages. It enables the creation of intricate shapes for precise fitting, crucial for tailored orthopedic implants^38^. By incorporating nanomaterials like graphene oxide, properties such as corrosion resistance and biocompatibility are significantly enhanced, ensuring durability and performance in physiological conditions. Moreover, powder metallurgy is cost-effective for large-scale production without compromising quality and meeting stringent medical standards. This integration paves the way for innovative medical devices, ultimately improving patient outcomes and advancing healthcare^4,39^. According to CDRH (the Center for Devices and Radiological Health-FDA), biocompatibility guidance could be impacted by metallic components in, on, or from a metallic device. It includes several tests to get permission for acceptable usage of metallic devices in patients that are considered by the FDA in the biological evaluation of implanted metals^40^.

Primary biological reactions around targeted tissues i.e. implementation, injections, and or/surgery need to be accurate investigations, not only to improve diagnoses but also to provide valid evidence for subsequent treatment^41–43^. The development of reliable experimental models for the clinical use of biomaterials and for predicting implant success or failure is becoming increasingly important in attaining adequate health and safety conditions. Animal models provide important biomaterial knowledge that eventually leads to the development of more effective clinical treatments for diseases in both humans and animals. Therefore, they act as a bridge between in vitro studies and in vivo clinical trials. The most commonly used laboratory animals have turned out to be rats, mice, and rabbits, probably because they are cheaper and easier to handle. However, no animal model presents the same anatomical, biochemical, physiologic, and biological characteristics as those found in human beings. Useful data for treating orthopedic patients are based not only on good planning and study design but also on perfect knowledge of the animal used and of the differences between the model and the human being. Regarding the in vivo test, there is no consensus about the appropriate animal model that can provide a clear indication of the systemic effects that metallic ions can cause in human beings^44^.

Therefore, the study aims to prepare bio-grade stainless steel powder with low amounts of elemental nickel and investigate the effects of incorporating graphene oxide (GO) in various proportions. These modifications will be carried out using powder metallurgy techniques to create nanocomposites. The study will focus on enhancing the properties of the material, including microstructural analysis, corrosion resistance, and biocompatibility to evaluate and compare the primary biological reactions to graphene oxide portions formulas in a designed Albino rats model.

## Materials and methods

### Synthesis of low-nickel bio-grade stainless steel samples

In this study, low-nickel bio-grade stainless steel powder (LNBGSS) was fabricated through the powder metallurgy method, a process known for producing high-quality metal components. The powder’s chemical composition was carefully controlled, consisting of 17% chromium (Cr), 0.5% nickel (Ni), 3% molybdenum (Mo), 10% manganese (Mn), 0.4% silicon (Si), 0.2% carbon (C), with the remaining balance being iron (Fe) as shown in Table 1. To ensure homogeneity, the stainless-steel powder was mixed and ground using a mortar and pestle for 30 min before the pressing stage. This manual grinding process helped to uniformly distribute the various alloying elements within the powder, enhancing the material’s overall properties.

**Table 1:** The composites of low-nickel bio-grade stainless steel powder (LNBGSS) with additive GO.

Sample’s name	Fe	Cr (%)	Ni (%)	Mo (%)	Mn (%)	Si (%)	C (%)	GO (%)
S0	Bal.	17	0.5	3	10	0.4	0.2	0
S1	Bal.	17	0.5	3	10	0.4	0.2	0.5
S2	Bal.	17	0.5	3	10	0.4	0.2	1.0
S3	Bal.	17	0.5	3	10	0.4	0.2	1.5

*Bal.* indicated the balance.

Subsequently, the powder was subjected to a compacting pressure of 800 MPa to form green compacts. This high pressure was essential to achieve a high density in the compacted samples, which is critical for the mechanical properties and overall quality of the final product. Before sintering, the green compacts underwent a preheating treatment at 600 °C in an inert N_2_ atmosphere. The compacted samples were then sintered at a temperature of 1050 °C for 2 h in an inert nitrogen (N_2_) atmosphere. Sintering at this temperature allows the powder particles to bond together through diffusion, resulting in a solid piece with improved mechanical strength and structural integrity^9,12^.

### Synthesis of graphene oxide (GO) samples

The synthesis of graphene oxide was carried out using a modified Hummers method, as detailed in our previous publication^45,46^. Pure graphite powder was employed as the starting material. The procedure began by mixing 46 mL of sulfuric acid (H_2_SO_4_) with 1.0 g of sodium nitrate (NaNO_3_) and 2 g of graphite powder. This mixture was then stirred in an ice bath for approximately 2 h. Then, 3.0 g of potassium permanganate (KMnO_4_) was gradually added to the mixture. After 2 h of continuous stirring, the solution began to turn brown. At this stage, 92 mL of distilled water was added drop by drop, followed by the rapid addition of another 150 mL of distilled water. To remove excess KMnO_4_ and halt the reaction, 20 mL of hydrogen peroxide (H_2_O_2_) was added slowly while stirring for 10 min. The resulting mixture was washed with 100 mL of a 5% hydrochloric acid (HCl) solution to further purify the product. To ensure the removal of all impurities, the mixture was repeatedly washed with distilled water and finally, 20 mL of acetone was added. The filtration of this mixture was then dried at 80 °C for 30 min to obtain graphene oxide in its powdered form^47^.

### Synthesis of low nickel bio-grade stainless steel (LNBGSS) powder with graphene oxide (GO)

The same synthesis method used for the preparation of low-nickel bio-grade stainless steel powder is applied to synthesize GO blended with bio-grade stainless steel. The GO was incorporated in different ratios. Specifically, three different concentrations of graphene oxide powder (0.5%, 1%, and 1.5%) were mixed with the stainless-steel powder using the powder metallurgy technique. For each concentration, the GO powder was blended with the low-nickel bio-grade stainless steel powder using a mortar and pestle. This step ensured a homogeneous distribution of graphene oxide within the stainless-steel powder. Subsequently, the blended powders were compressed using an appropriate die to form compacted samples. The green compact sample was preheated at 600 °C in an inert N_2_ atmosphere to stabilize the GO-derived structure**.** Finally, all samples, including the low-nickel bio-grade stainless steel powder sample without graphene oxide, were sintered at 1050 °C in the N_2_ atmosphere.

### Preparation of injectable solution

Each sample of 20.5 mg was weighted and then dissolved in phosphate-buffered saline solution (PBS) to be adjusted to the desired target concentration of 200 ug/L using PBS. The target concentration of 200 ug/L was based on a study in which the metal ion level of a joint puncture in patients was analyzed before revision surgery, which showed average concentrations in the range of 200–250 ug/L in the joint^48,49^.

### Animals used in the study

Thirty-six albino rats, weighing between 165 and 195 g, were obtained from the animal house of the Egyptian Drug Authority (EDA), Egypt (AEC 2354), to establish an animal model for evaluating biological reactions. The rats were randomly distributed to six groups (n = 6): phosphate-buffered saline buffer control group (SB), graphene oxide group (SGO), low-nickel bio-grade stainless steel powder (LNBGSS) group (S0), and three groups of LNBGSS powder mixed with different concentrations of graphene oxide: 0.5% (S1), 1.0% (S2), and 1.5% (S3). All experimental steps involving animals were performed according to the rules and regulations of the Animal Protection Laboratory Animal Regulations^50^.

### Intra-articular injection

The sonication of all solutions occurred for 60 min before the intra-articular injection to avoid any possible aggregations^50^. Under sterile conditions, 100 uL of all prepared suspension solutions were injected into the left knees of all groups of rats. After 10 days incubation period, animals were re-weighted, then, anesthetized by thiopental, and then euthanized by separation of the spinal cord (animal house, EDA, Egypt)^49,50^.

### Blood collection

Blood samples were collected from each group for analysis. Samples for a complete blood count were collected into ethylene diamine tetraacetate (EDTA)–treated tubes. Samples for biochemical analysis were collected into EDTA-free test tubes. These blood samples for biochemical analysis were centrifuged for 15 min to separate the serum. Subsequently, the samples were analyzed for both hematological and biochemical parameters^51^.

### Hematological parameters

Complete blood count (CBC): including hemoglobin (HB), white blood cells (WBC), red blood cells (RBC), platelets, monocytes, mean corpuscular volume (MCV) and mean corpuscular hemoglobin concentration. Each mentioned item has a normal range^52^.

### Blood biochemical indices

Liver enzymes: total protein (TP), alanine transaminase (ALT), aspartate transaminase (AST), and total bilirubin (TB); kidney functions: (urea nitrogen (UN), serum creatinine (SCR))^53^.

### Statistical analysis

The data are expressed as the mean ± standard deviation (SD). The statistical significance was assessed by the one-way analysis of variance (ANOVA) test followed by the Tukey test, enabling multiple comparisons for all tests using SPSS 19.0 (SPSS Inc., Chicago, USA). A *P* value of less than 0.05 (*P* < 0.05) was considered statistically significant.

### Characterization techniques

The X-ray diffraction (XRD) patterns of all prepared samples were obtained using a Malvern Panalytical Empyrean 3 diffractometer. Phase structure analysis was performed with a Bruker D8 Advance diffractometer, utilizing Copper K_α_ radiation (wavelength = 1.54056 Å) at an operating voltage of 40 kV and a current of 40 mA. The samples were scanned in the 2θ range of 10°–80° in step-scan mode, with a step size of 0.02°. Field emission scanning electron microscopy (FE-SEM) images were captured using an FEI Quanta 200 microscope, operated at 20 kV. The electrochemical measurements for corrosion analysis were performed using METROHM (Autolab PGSTAT 302N) potentiostat controlled by NOVA software for precise data acquisition and analysis. The experiments were conducted in a 3.5% NaCl aqueous solution, chosen to simulate a physiological corrosive environment similar to human blood plasma. The pH of the electrolyte was measured before each experiment and maintained within the range of ~ 6.5 to 7.0, closely resembling the pH of the blood. Electrochemical impedance spectroscopy (EIS) was employed to assess the electrochemical behavior of the samples over a frequency range from 100 kHz to 10 mHz. A sinusoidal AC voltage signal with an amplitude of 10 mV (rms) was applied to perturb the system, allowing for the analysis of charge transfer resistance, corrosion kinetics, and interfacial properties of the material in a biologically relevant environment. Liver function and kidney function were measured using a Semi-auto biochemistry analyzer. A complete blood picture was measured by CBC analyzer Derui BCC_3600.

## Results and discussions

### X-ray diffraction patterns

**Fig. 1:**
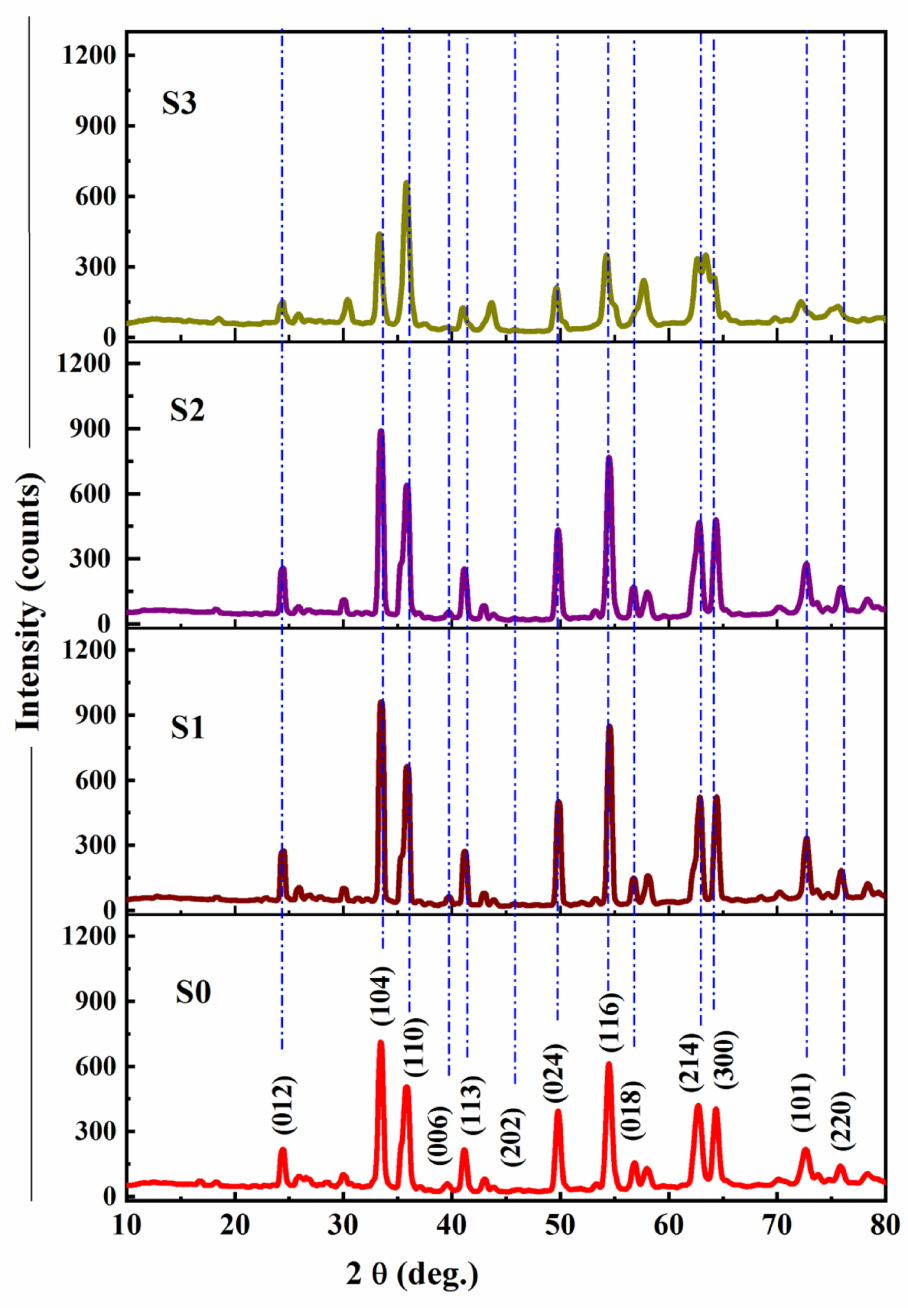
X-ray diffraction patterns of (S0) pure low-nickel bio-grade stainless steel powder (LNBGSS), (S1) LNBGSS with 0.5 wt% GO, (S2) LNBGSS with 1.0 wt% GO, and (S3) LNBGSS with 1.5 wt% GO.

The XRD patterns for GO incorporated at ratios of 0.5, 1.0, and 1.5 wt% were examined and compared with the XRD graph of pure low-nickel bio-grade stainless steel powder LNBGSS as shown in Fig. 1. The XRD pattern of the S0 sample exhibits well-defined diffraction peaks at various 2θ positions corresponding to specific crystal planes, different peaks. These peaks confirm the presence of α-Fe_2_O_3_ (hematite) as the primary crystalline phase, in agreement with the standard JCPDS No. 33-0664^54^. The sharp and intense peaks observed in S0 indicate a well-crystallized hematite structure. Upon incorporating GO into the LNBGSS matrix, the XRD patterns of S1 and S2 exhibit similar diffraction features to S0, with no significant shifts in peak positions. This suggests that the hematite phase remains dominant and structurally stable at lower GO concentrations (0.5 and 1.0 wt%). However, in the S3 sample (1.5 wt% GO), noticeable changes occur in the diffraction pattern. The peaks become broader and may result from the interaction between GO and Fe_2_O_3_, leading to structural distortions and possible strain within the crystal lattice.

Interestingly, two distinct peaks were observed at 2θ = 62.7° and 64.2°, corresponding to the (214) and (300) planes, respectively. With increasing GO content, these peaks merged into a single peak at a higher doping level of 1.5 wt% GO. In the S0, S1, and S2 samples, these peaks remain distinct. However, in the S3 sample, these peaks merge into a single broader peak, suggesting an alteration in the crystal structure, possibly due to increased strain, defect formation, or phase interaction between GO and hematite. This merging effect at higher GO concentrations may be attributed to incorporating GO functional groups into the hematite lattice, which modifies the local atomic arrangement. Moreover, the intensity of the peak at 2θ = 73° initially increased with small amounts of GO but then decreased at 1.5 wt% GO content. This behavior was also noted for the dominant peaks of the hematite phase (α-Fe_2_O_3_) at 33.2°, 35.6°, and 54.1°, whereas the intensity of the (110) peak increased with increasing GO content, even at 1.5 wt%. These observations suggest that at high GO content, graphene sheets progressively covered the composite surfaces, leading to a decrease in peak intensity for most planes except the (110) plane.

Additionally, the S3 sample shows an increased intensity in the (110) plane, suggesting a preferential orientation along this crystallographic direction. This enhancement in intensity could indicate that GO incorporation influences the grain growth mechanism during synthesis or sintering, leading to a preferential alignment of hematite grains along the (110) plane. The presence of GO may selectively stabilize this plane due to charge transfer effects or bonding interactions between GO functional groups (such as –COOH and –OH) and Fe_2_O_3_. The reduction in crystallinity observed in the S3 sample is likely due to the incorporation of large graphene sheets within the structure^55^. These sheets interact with the hematite matrix, introducing strain and molecular disorder, which can alter the nucleation and growth of crystalline phases. Furthermore, the formation of new chemical bonds between GO and Fe_2_O_3_ may contribute to lattice distortions, affecting peak broadening and intensity variations^56,57^.

### Field-emission scanning electron microscopy (FE-SEM)

**Fig. 2:**
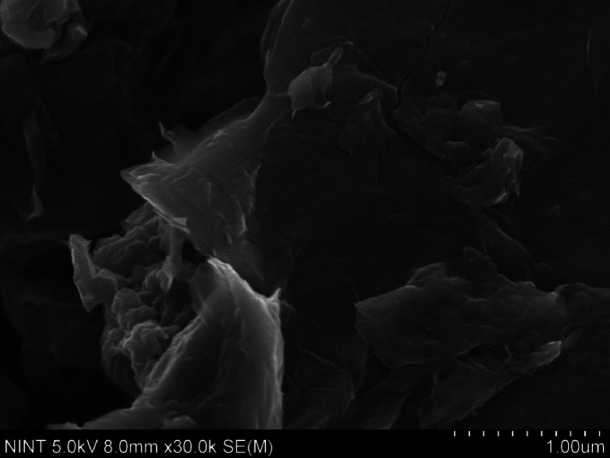
FE-SEM image of graphene oxide (GO).

The morphology and microstructures of stainless-steel matrix composites incorporated with graphene oxides prepared using the metallurgy technique, were investigated using FE-SEM. Figure 2 depicts the microstructures of graphene oxide (GO) powder. The FE-SEM image reveals the characteristic wrinkles in the graphene oxide sheets, indicating their typical morphology. Additionally, the presence of large sheets at the microscale further confirms the high quality of the graphene oxide preparation. These features suggest that the GO was successfully synthesized with a well-defined structure, maintaining the integrity and desirable properties of graphene oxide, such as high surface area and mechanical strength^58^.

**Fig. 3:**
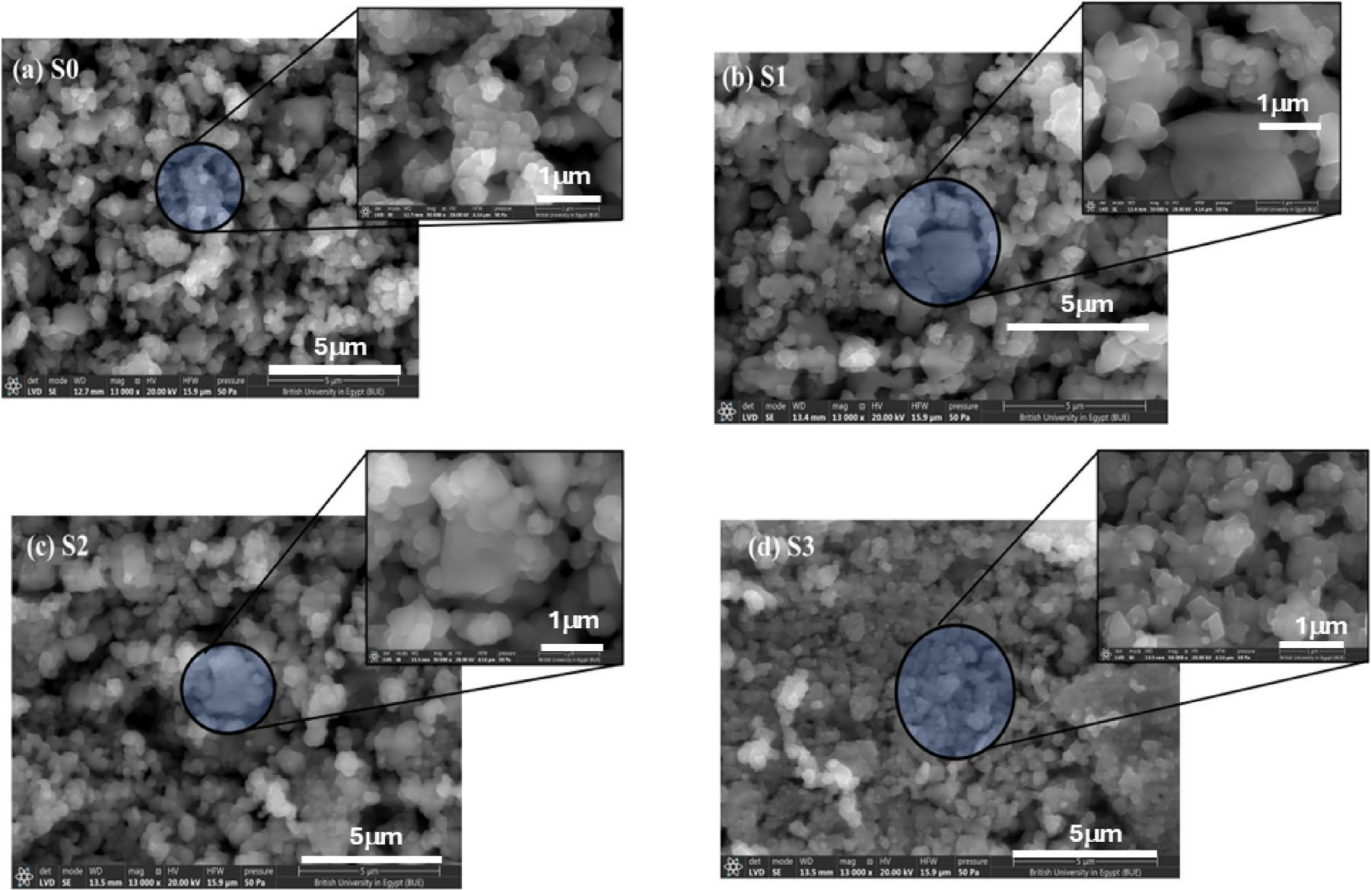
(**a**–**d**) FE-SEM images of LNBGSS incorporating different GO content, (**a**) zero GO, (**b**) 0.5 wt% GO, (**c**) 1.0 wt% GO, and (**d**) 1.5 wt% GO. For each picture, there are two magnifications.

Figure 3a–d shows the FE-SEM images of composites incorporating different wt% of GO into low-nickel bio-grade stainless steel (LNBGSS). Figure 3a presents the LNBGSS with zero wt% of GO. The FE-SEM images at various magnifications reveal almost uniformly shaped particles with some small aggregations, indicating the homogeneity and consistent quality of the LNBGSS sample without GO incorporation. The uniform particle distribution suggests that the base stainless-steel matrix was well-prepared and suitable for further composite formation. Besides, adding 0.50 and 1.0 wt% of GO to LNBGSS changes its microstructure as seen in Fig. 3b and c. Even at these small incorporation amounts of GO, an increase in particle aggregation is evident. This aggregation can be attributed to the interaction between the GO sheets and the stainless-steel particles, which may lead to enhanced bonding and interfacial properties in the composite. At 1.5 wt% of GO content (Fig. 3d), the microstructure undergoes a notable transformation. The GO sheets are seen to cover the LNBGSS particles extensively, resulting in a composite where the particles maintain similar shapes but vary in size. The extensive coverage by the GO sheets indicates effective dispersion and integration of GO within the stainless-steel matrix. This integration is crucial as it could improve the composite properties.

**Fig. 4:**
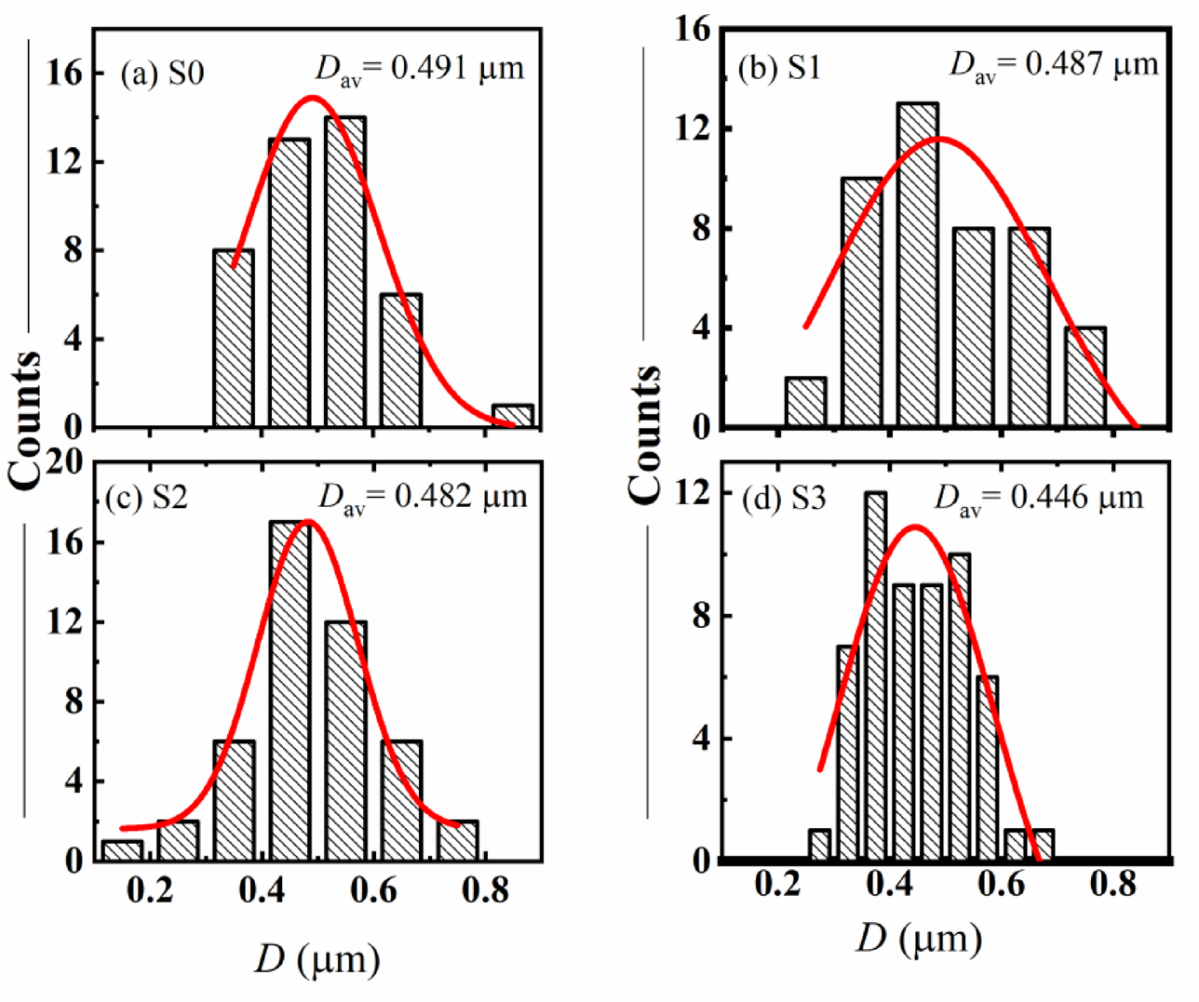
(**a**–**d**) Histograms for particle size distribution of LNBGSS and GO/LNBGSS composites.

The FE-SEM analysis reveals that GO incorporation significantly influences the morphology and particle size distribution of LNBGSS composites. At lower GO concentrations (0.5 and 1.0 wt%), GO improves dispersion while remaining physically embedded. However, at higher GO content (1.5 wt%), GO interacts strongly with the matrix, resulting in finer particles, altered grain structure, and possible chemical integration. These findings highlight the role of GO in tailoring microstructure, which could have implications for the mechanical and functional properties of the composite materials. The average particle sizes (*D*_av_) were calculated for all studied samples, as shown in Fig. 4a–d. It was found that *D*_av_ slightly decreased with increasing the content of GO. It ranges from 0.491 µm for S0 to 0.466 µm for S3.

### The electrochemical corrosion characteristics

#### Potentiodynamic polarization measurements

**Fig. 5:**
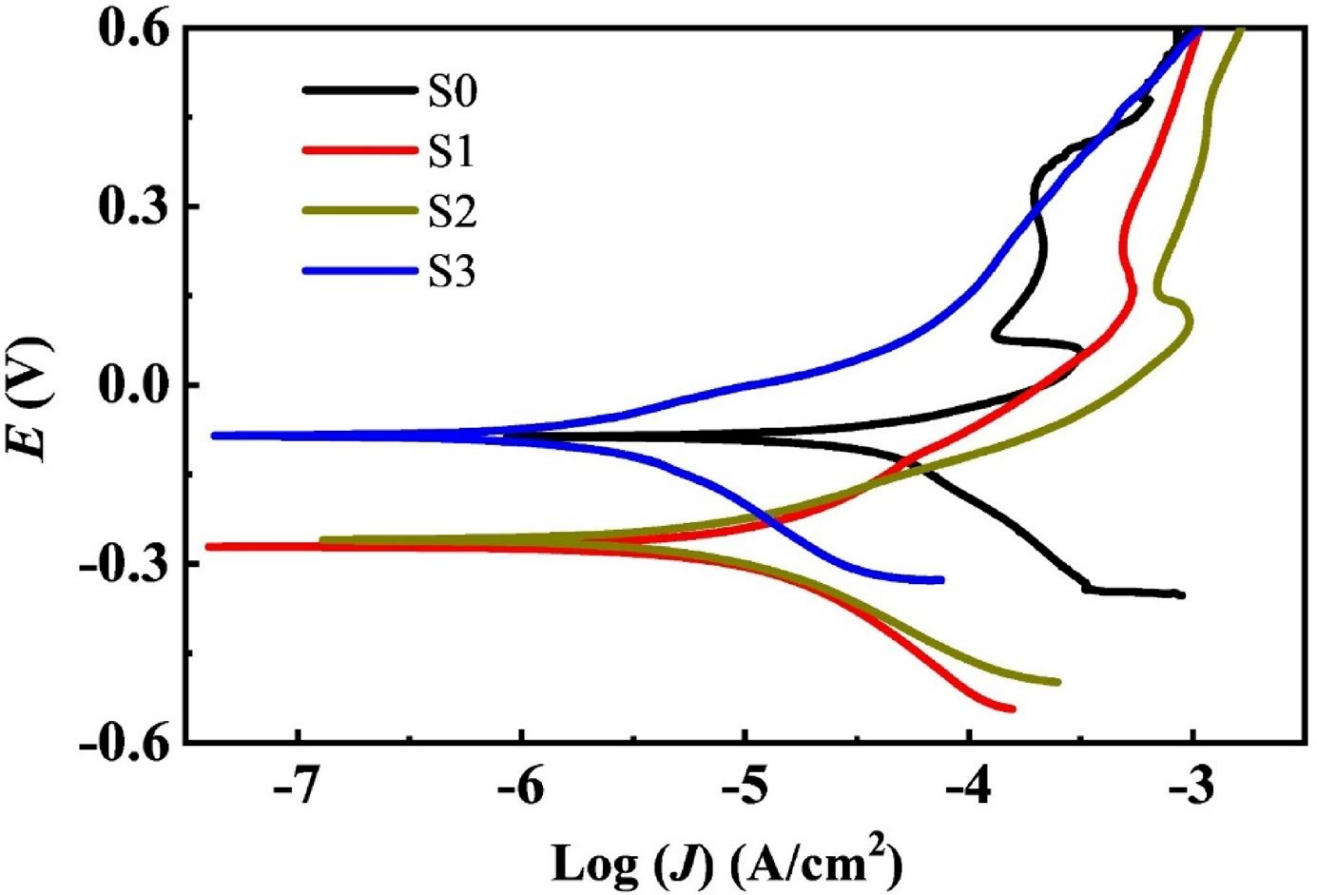
Tafel curves of LNBGSS incorporating different GO content.

The electrochemical corrosion test of low nickel bio-grade stainless steel (LNBGSS) and the composites with graphene oxide (GO) additives was conducted in a solution with the same pH value as hemoglobin or a potentially corrosive environment. The linear sweep voltammetry (LSV) curves for LNBGSS and the impact of the GO additives on the composites are illustrated in Fig. 5. The Tafel polarization technique is a systematic and effective approach to examine the electrochemical activity of electrocatalysts. Using a three-electrode system, the data were recorded from − 0.3 to 1 V versus Ag/AgCl (reference electrode) at a scan rate of 200 mV/s in 3.5% NaCl.

As shown in Fig. 5, the Tafel polarization behavior of LNBGSS is affected by the GO additives. From the Tafel polarization curves, corrosion parameters such as corrosion current density (*J*_corr_), corrosion potential (*E*_corr_), anodic Tafel slope (*β*_a_), and cathodic Tafel slope (*β*_c_) were derived and listed in Table 2. The used Tafel relation is^59^:

1 $$J={J}_{corr}\text{exp}\left(\frac{2.303 E-{E}_{corr}}{{\beta }_{a}}\right)-\text{exp}\left(\frac{2.303E-{E}_{corr}}{{\beta }_{c}}\right)$$

where *J*_corr_ is the current density at the corrosion potential (*E*_corr_). *β*_a_ and *β*_c_ are the anodic and cathodic slopes. *J* and E are the current density and the applied potential*,* respectively. Table 2 presents the corrosion parameters for electrochemical polarization measurements of LNBGSS composites with GO additives.

**Table 2:** Listed are the kinetic parameters of the prepared samples: the corrosion rate, corrosion potential, *E*_corr_, density, *J*_corr_, anodic Tafel slope, *β*_a_, and cathodic Tafel slope, *β*_c_.

Sample’s name	Corrosion rate (mm/year)	*E*_corr_ (V)	*J*_corr_ (A/cm^2^)	*β*_c_ (V/dec)	*β*_a_ (V/dec)	*η*_*p*_ %
S0	1.773	− 0.086	1.30 × 10^−4^	0.238	0.115	–
S1	0.364	− 0.271	2.40 × 10^−5^	0.225	0.208	81.5
S2	0.207	− 0.26	1.27 × 10^−5^	0.182	0.113	90.2
S3	0.031	− 0.085	1.89 × 10^−6^	0.203	0.091	98.5

The inhibition efficiency according to the potentiodynamic polarization measurements (*η*_p_%) of the composites was also calculated.

*Bal*. indicated the balance.

The values of *J*_corr_ for LNBGSS composites with GO additives significantly decreased compared to the LNBGSS composites without GO, as seen in Table 2. This reduction in corrosion current density indicates the effectiveness of the GO additive in enhancing corrosion protection. Specifically, the corrosion current density of the LNBGSS composite without GO was 1.3 × 10^−4^ A/cm^2^, while with GO additives, it was reduced to 2.4 × 10^−5^ A/cm^2^ for sample S1, 1.27 × 10^−5^ A/cm^2^ for sample S2, and 1.89 × 10^−6^ A/cm^2^ for sample S3. Besides, the average corrosion rate was 1.773 mm/year for the composite without GO. Adding GO significantly decreased the rates to 0.364, 0.207, and 0.031 mm/year for composites with 0.5, 1.0, and 1.5 wt% of GO additives, respectively. Moreover, both *β*_a_ and *β*_c_ were altered by adding Go to LNBGSS. It would be useful to calculate the inhibition efficiency (*η*_p_%) for the studied composites as^60^:

2 $${\eta }_{\text{p}}\mathbf{\%}=\frac{({J}_{o}-J)}{{J}_{o}} \times 100$$

where *J*_o_ is the current density without an inhibitor, while *J* is the value with an inhibitor. As seen from Table 2, the addition of GO to LNBGSS composites significantly increased the values of *η*_p_ %, indicating a higher corrosion resistance.

**Fig. 6:**
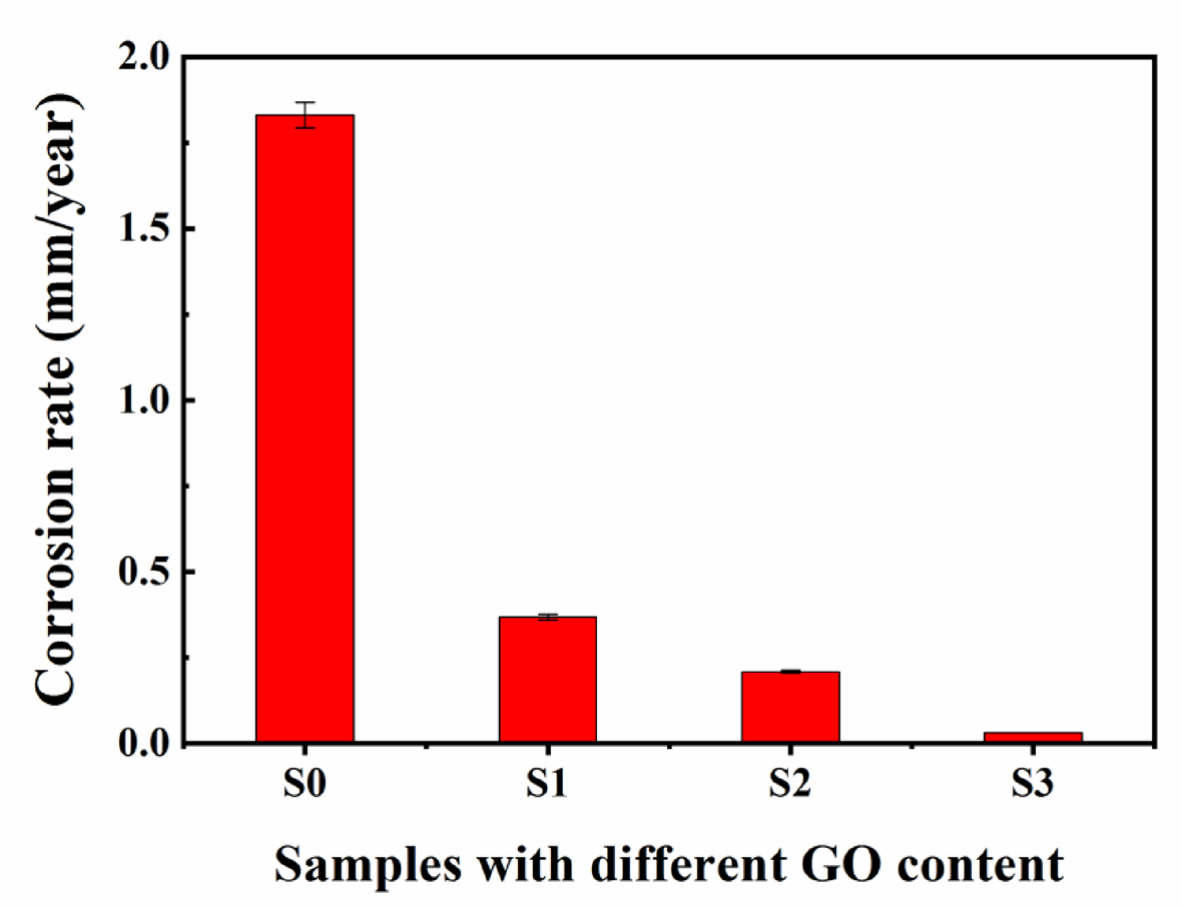
The average corrosion rates of low nickel bio-grade stainless steel (LNBGSS) incorporating different GO content.

Figure 6 illustrates the average corrosion rates of LNBGSS with GO nanoparticles added. Notably, the 0.5 wt% GO additive reduced the electrochemical corrosion rate by 81.5% compared to the sample without GO. Increasing the GO content to 1.0 and 1.5 wt% further decreased the electrochemical corrosion rate by 90.2% and 98.5%, respectively. These results demonstrate the substantial impact of GO additives on the corrosion resistance of LNBGSS composites.

#### Electrochemical impedance measurements

**Fig. 7:**
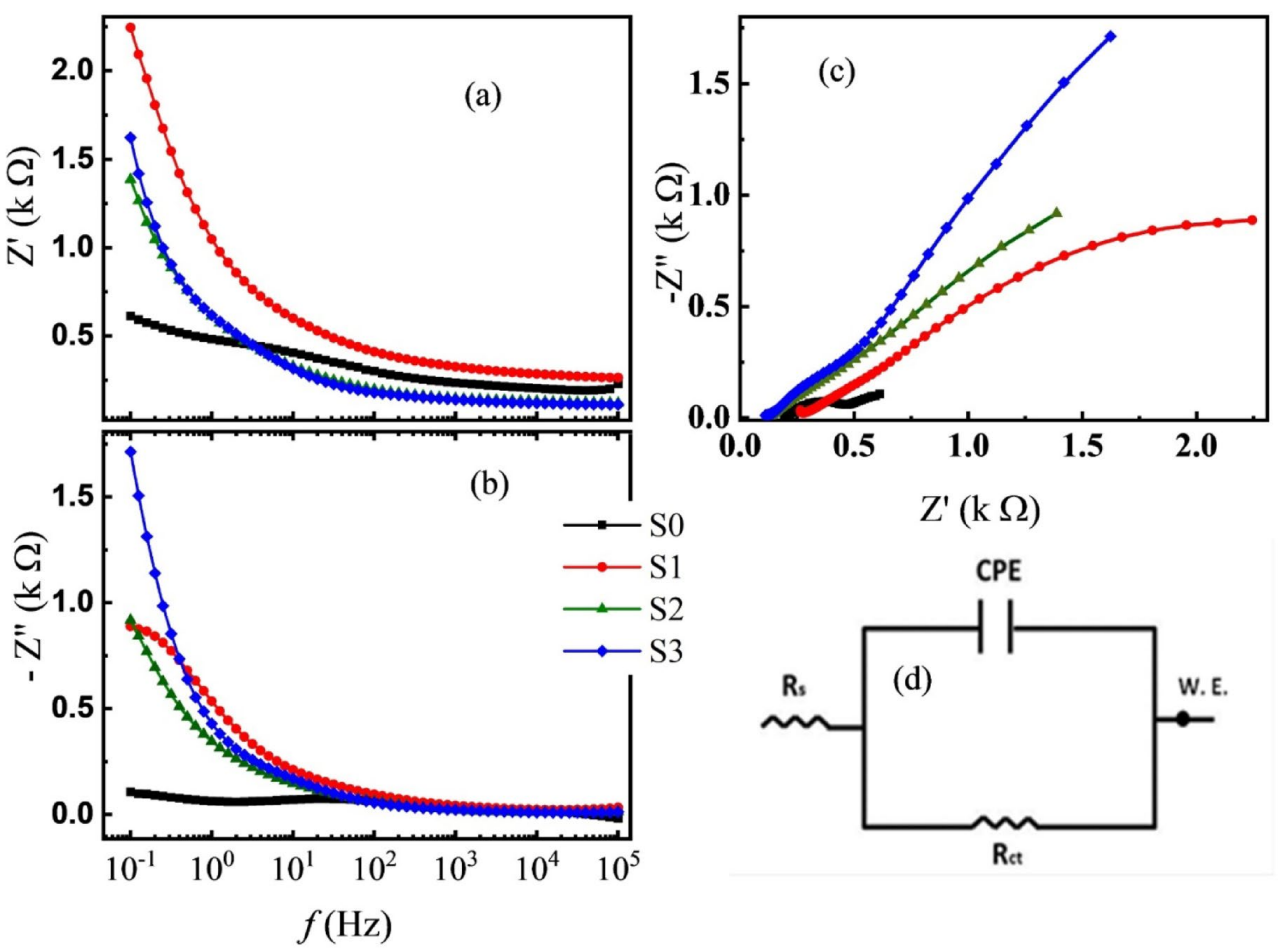
(**a**–**d**) Both (**a**) and (**b**) showed the change of the real and the imaginary parts of the impedance with frequency, respectively, (**c**) Nyquist plots at room temperature, and (**d**) the suggested one, an equivalent circuit model.

The electrochemical impedance spectroscopy is a powerful technique for investigating electrochemical and corrosion systems. For impedance measurements, an AC superimposed 10 mV amplitude signal peak to peak is normally used. The working frequency range is 0.1–10^5^ Hz. The measurements, processing, storage, retrieval, and analysis of data have been automated. After preliminary setting-up measurements, the recorded impedance data were recorded over the specified frequency range and displayed in Fig. 7a and b. Both Z′ and Z″ decreased with increasing frequency owing to the lag between the electric dipoles and the applied field. The experimental impedance data could be approximately explained by one of the suggested equivalent circuit models (see Fig. 7d). The electrochemical impedance data are given in Table 3. Generally, it was found that the polarization resistance or charge transfer, R_ct_, of the LNBGSS composite increases as the wt% ratio of GO increases.

**Table 3:** The electrical parameters for electrochemical corrosion experiments and the inhibition efficiency based on electrochemical impedance measurements (*η*_*e*_%) for the composites LNBGSS with the additive of GO.

Sample’s name	*R*_ct_ (k Ω) polarization resistance	*R*_s_ (Ω) solution resistance	CPE.Y_0_ (F)	*η*_e_%
S0	0.811	0.174	1.30 × 10^−3^	–
S1	4.290	0.322	3.70 × 10^−4^	81.1
S2	37.46	0.116	4.24 × 10^−5^	97.8
S3	98.30	0.154	1.61 × 10^−6^	99.2

The value of the R_ct_ of the LNBGSS composite without GO was measured at 0.811 kΩ. With the addition of GO, the polarization resistance values increased markedly: 4.29 kΩ for the composite with 0.5 wt% GO, 37.46 kΩ for the composite with 1 wt% GO, and 98.3 kΩ for the composite with 1.5 wt% GO. These results demonstrate that the incorporation of GO into the LNBGSS composites substantially enhances their corrosion resistance. At the same time, the small Rs value confirms that there are no errors in the corrosion rate calculations. Additionally, the values of the constant phase element in the equivalent circuit, CPE.Y0(F), decreased as well with the addition of GO which indicates improvement of the surface properties and better corrosion resistance. This is a further indication of improving the electrochemical properties and reduced corrosion activity in the LNBGSS composites with GO^61^. The values of *η*_e_% were also calculated (see Eq. 3) and listed in Table 3.

3 $${\eta }_{\text{e}}\mathbf{\%}=\left(\frac{\left({\text{R}}_{\text{ct}}\right)inh-{\text{R}}_{ct}}{{(\text{R}}_{ct})inh}\right)\times 100$$

where (*R*_ct_)inh and *R*_ct_ are the resistances of charge transfer or polarization resistance with inhibitor and without inhibitor, respectively.

One noted that there is a good matching between the estimated η % by this method and that by polarization one. Besides, Fig. 7c depicts the Nyquist plots of the studied composites at room temperature. Increasing the impedance, i.e. inhibitor resistance of LNBGSS with increasing the concentration of GO.

### General health status of animals

**Fig. 8:**
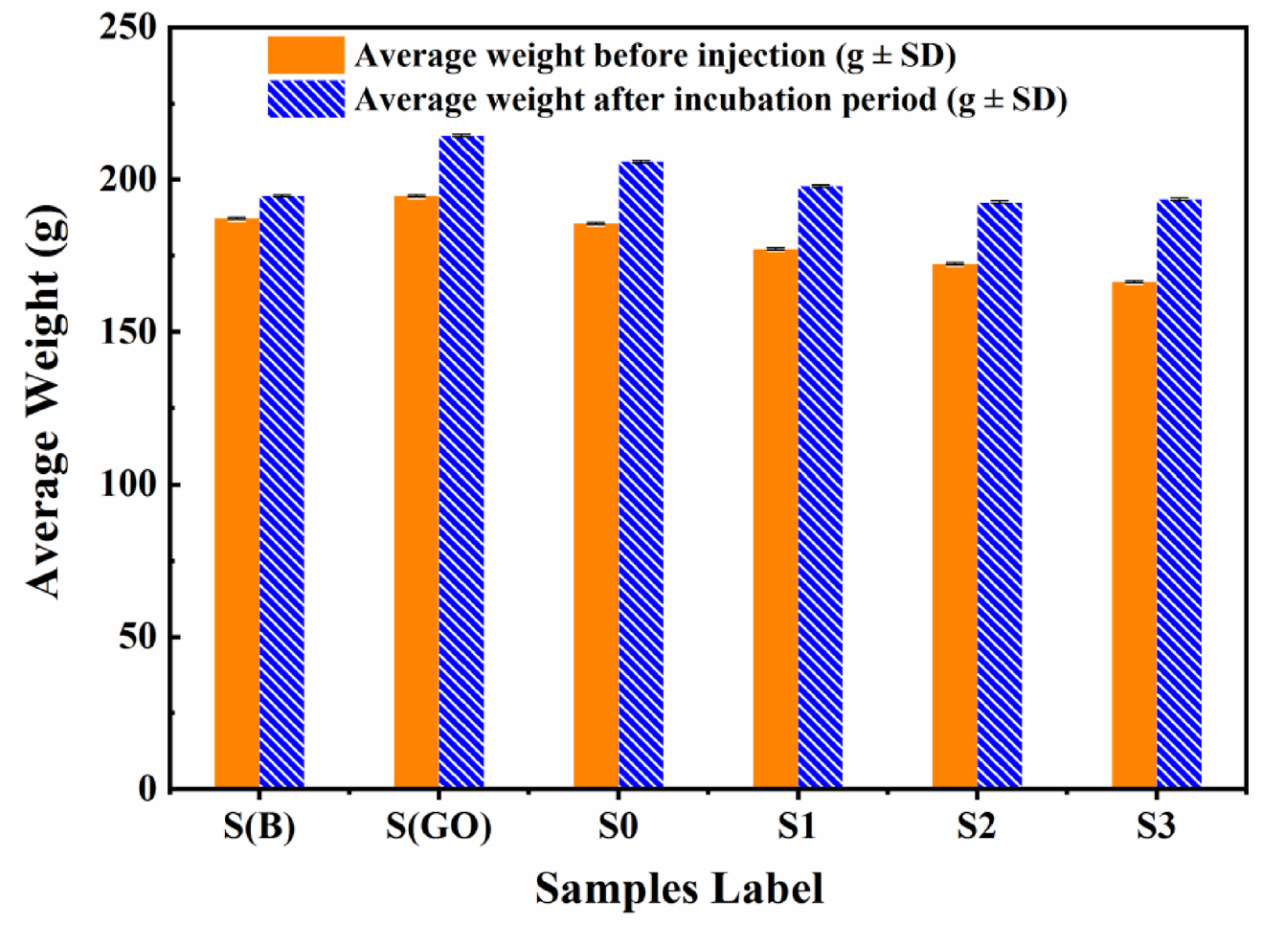
Average body weight of control, graphene oxide (GO), LNBGSS, and LNBGSS samples with varying concentrations of GO before and after injection.

Monitoring the health of animals, including no death cases recorded, vital signs, mobility, and signs of pain or discomfort, is an essential requirement for assessing the effects of substances injected into the synovial layer of the left knee during the 10 days. This monitoring provides a clear evaluation of the injected material’s impact on the animals, including potential systemic effects, inflammatory responses, and infection risks. It was observed that none of the animal groups exhibited significant physical or behavioral changes, such as fever, hair loss, oedema, or swelling. However, animals in group S0 showed slight oedema at the knee injection site. Furthermore, all groups demonstrated a slight increase in body weight (BW) during the incubation period, as shown in Fig. 8, with no significant differences between the groups.

It is indicated that no death cases were recorded, and no major physical or behavioral changes were observed, despite slight oedema noted in animals injected with low-nickel bio-grade stainless steel powder (LNBGSS) in group S0. The observed body weight gain suggests that graphene oxide at different concentrations is biocompatible and safe for intra-articular injection into the knees and did not exhibit obvious toxicity to rat^17,62^.

### Hematological parameters

**Fig. 9:**
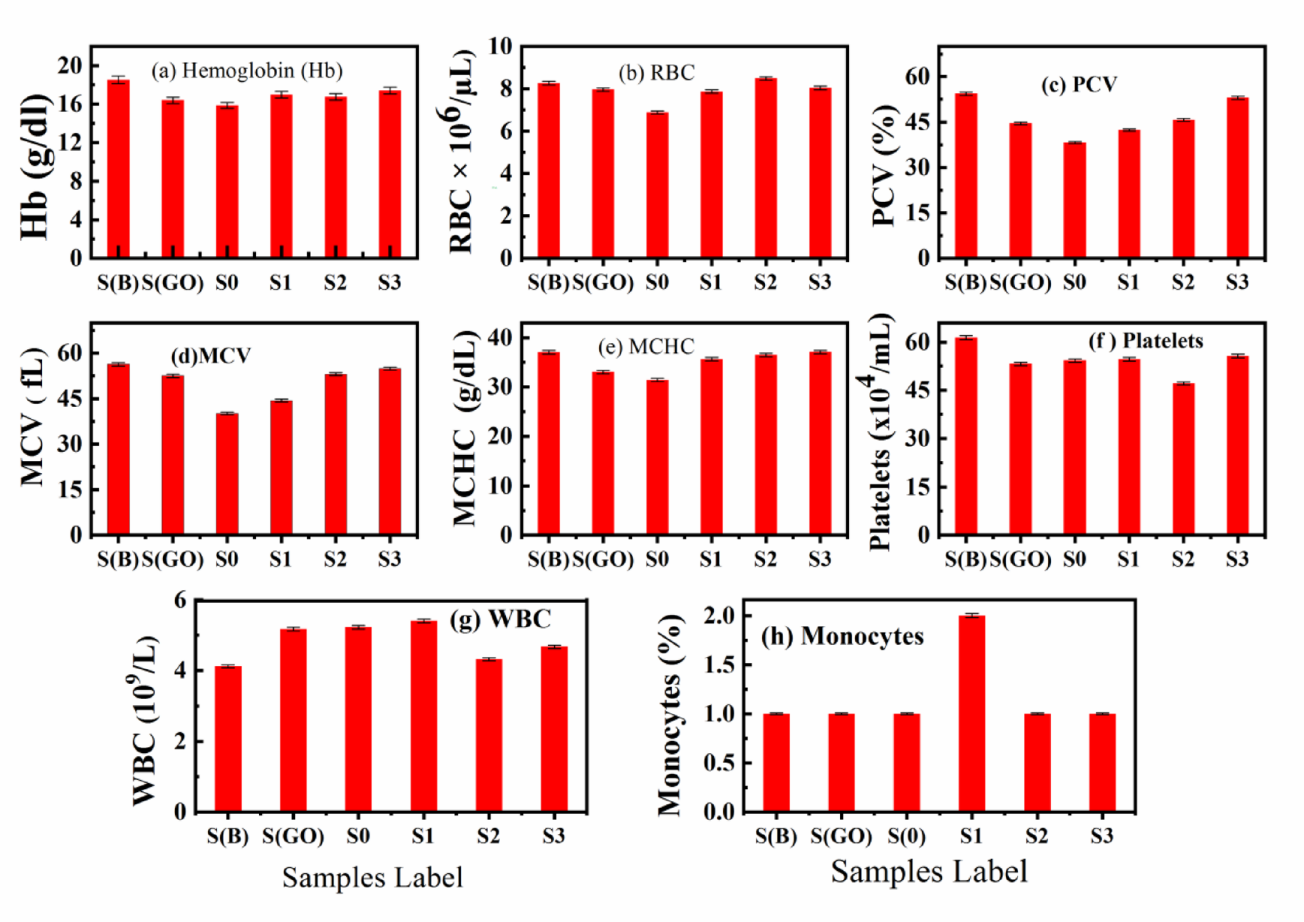
(**a**–**h**) Complete blood count (CBC) of different Samples; (**a**) Hemoglobin (HB), (**b**) Red blood cells (RBC), (**c**) Packed cell volume (PCV), (**d**) Mean corpuscular volume (MCV), (**e**) Mean corpuscular hemoglobin concentration (MCHC), (**f**) Platelets, (**g**) White blood cells (WBC), and (**h**) Monocytes.

Figure 9a–h displays the complete blood count (CBC) results for various samples, important critical hematological parameters. These parameters are essential indicators of an organism’s health, offering valuable insights into both physiological and pathological conditions. Typically, hematological assessments include measurements of hemoglobin (HB), red blood cells (RBC), packed cell volume (PCV), mean corpuscular volume (MCV), mean corpuscular hemoglobin concentration (MCHC), platelet count, white blood cells (WBC), and monocyte count. It found that no significant deviations in hematological parameters were observed when comparing the test samples to the control sample, as depicted in Fig. 9. However, an analysis of the low-nickel bio-grade stainless steel powder (LNBGSS) sample (referred to as S0) revealed distinct variations in several parameters: the PCV was 38.216%, RBC count was 6.88 × 10^6^/μL, MCV was 40.08 fL, MCHC was 40.08 g/dL, and HB was 15.86 g/dL. Although these values represented the lowest among all the samples tested, they still fell within the normal reference ranges^63^, affirming the suitability of this sample for use as bio-graded steel.

The hematological data for all injected rat samples fell within the normal ranges according to the references^52,64^. Notably, while the rats injected with the LNBGSS, i.e. S0 sample exhibited the lowest hematological values among all examined groups, the use of GO in other samples appeared to enhance the characteristics as the concentration of GO increased. This suggests that no anaemia was induced by the potential toxicity of the nanomaterials used for biomedical applications in these investigated rats. It is crucial that nanomaterials intended for biomedical applications demonstrate compatibility with blood and do not cause hemolysis or abnormalities in hematological parameters when tested in vivo^65–67^.

### Biochemical serum parameters

In the current study, liver function was assessed by measuring serum levels of AST (aspartate aminotransferase), ALT (alanine aminotransferase), total protein, and total bilirubin. These enzymes are critical indicators of drug-induced liver toxicity in laboratory animals. They are primarily found in hepatocytes, where they facilitate the conversion of amino acids into glutamate through the transfer of amino groups^68^. Therefore, elevated levels of these enzymes in the bloodstream are among the earliest laboratory signs of hepatic dysfunction.

**Fig. 10:**
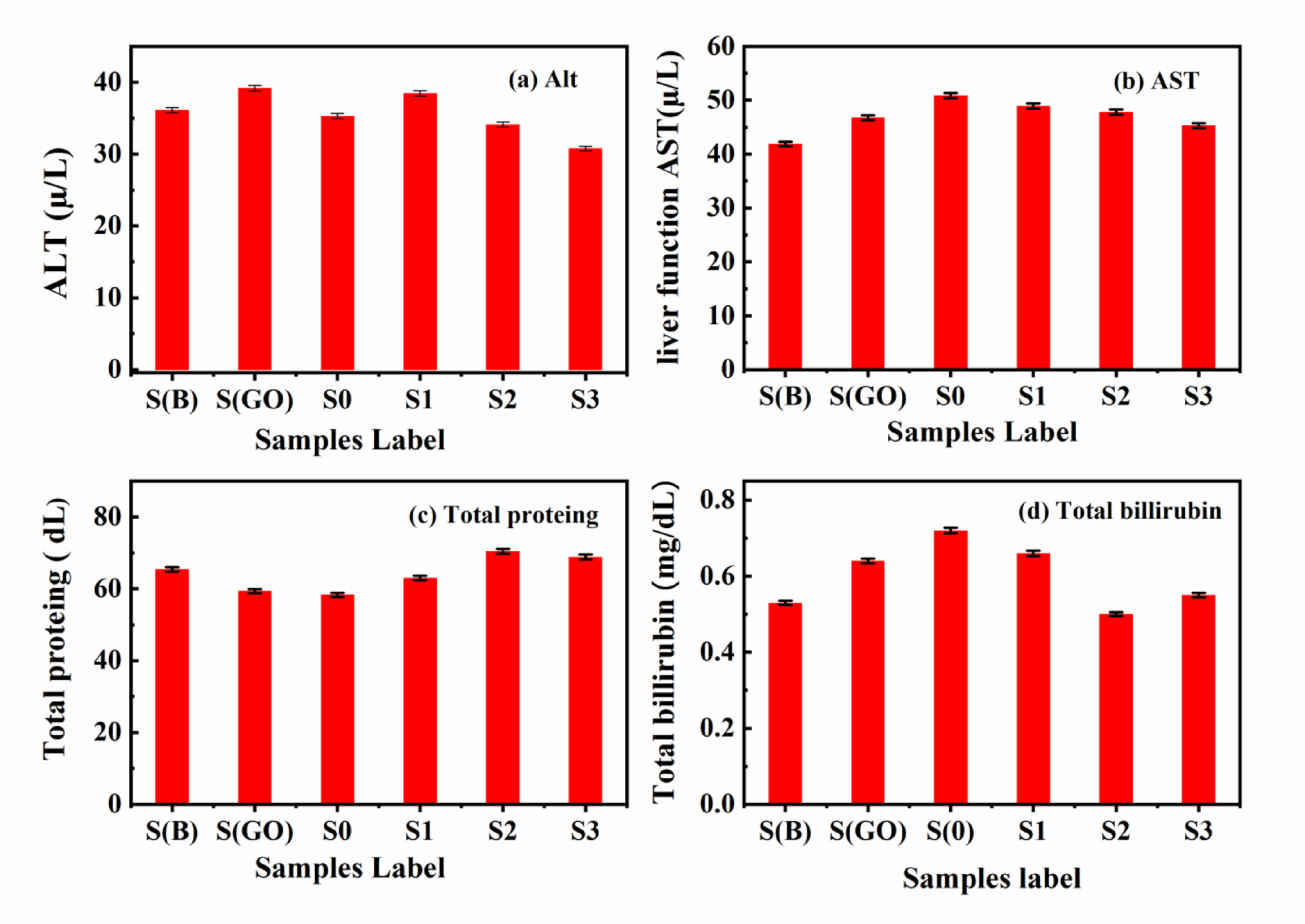
(**a**–**d**) Liver function tests of samples from different groups; (**a**) ALT, (**b**) AST, (**c**) Total protein, and (**d**) Total bilirubin.

**Fig. 11:**
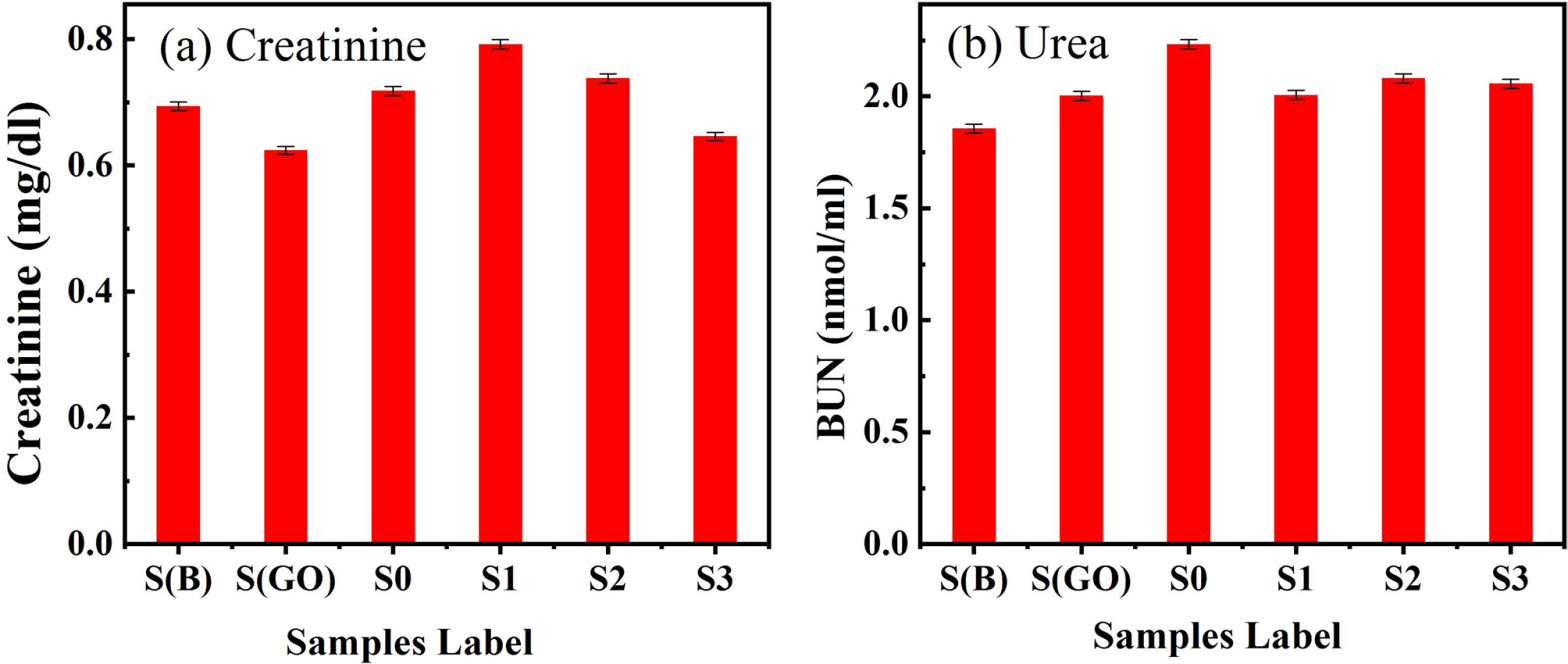
(**a**,**b**) Kidney function tests of samples from different groups; (**a**) creatinine, (**b**) urea.

As illustrated in Fig. 10a–d, no significant changes were observed in enzyme levels compared to the control samples group throughout the scheduled incubation period. According to reference data, all examined groups exhibited enzyme levels within the normal range, with no significant differences noted (*P* ≥ 0.05). This suggests that the use of graphene oxide (GO) did not produce any abnormal or toxic effects on liver function. Additionally, renal glomerular function was evaluated by measuring creatinine and blood urea nitrogen (BUN) levels^69^. No significant alterations in either BUN or creatinine levels were observed in all injected rats compared to the control group, with results supporting a significance level of (*P* < 0.05), as seen in Fig. 11^70^.

BUN analysis provides valuable insights into liver function, as nitrogen from ammonia produced by the liver contributes to urea formation, which is then excreted by the kidneys as a metabolic waste product^67^. Elevated BUN levels may indicate dysfunction in either the kidneys or the liver. In humans, low BUN levels have been associated with various conditions, including trauma, surgery, malnutrition, opioid and anabolic steroid use, and fluid overload^71^. However, low BUN levels are relatively uncommon and rarely reported in animals. According to the normal hematological parameters and biochemical indicators, graphene oxide nanoparticles are considered safe nanoparticles^70,71^.

The possible mechanism of GO’s effects on rats is suggested: upon injection, GO directly enters the bloodstream and, as a foreign substance, is recognized and tracked by immune cells. It rapidly distributes to the lungs, liver, spleen, and kidneys but does not cross the blood–brain barrier. At the administered doses, these organs, such as the liver, spleen, and kidneys can tolerate GO and maintain normal function without significant changes. However, the precise interactions between GO and immune cells in vivo remain unclear and require further investigation^72^.

## Conclusions

This study demonstrates incorporating graphene oxide (GO) into low nickel bio-grade stainless steel (LNBGSS). Most of the XRD peaks indicated the existence of a hematite phase (α-Fe_2_O_3_) structure. Because of the crosslinking between LNBGSS and the GO sheets, some of the XRD characteristic peak intensity decreased, and a reduction in crystallinity could exist. FE-SEM images revealed the effective dispersion of GO within the LNBGSS matrix. Besides, the integration of varying GO concentrations (0.5, 1.0, and 1.5 wt%) within the LNBGSS matrix caused a reduction in the corrosion rates achieving a 98.5% decrease for the highest GO concentration. It is also found that the addition of GO to LNBGSS composites significantly increased the polarization resistance, indicating a higher corrosion resistance. It was found that the calculated inhibition efficiency increased markedly with increasing the content of GO in LNBGSS composites.

Importantly, in vivo biocompatibility assessments in albino rats showed no significant adverse effects on hematological parameters of the animal model, liver enzymes, or kidney functions, indicating that the modified composite is biologically safe. Furthermore, no toxic effects on the general health status, growth, overall appearance, and body weight gain. These findings suggest that GO-modified LNBGSS is a promising material for applications requiring both enhanced corrosion resistance and biocompatibility, making it suitable for use in biomedical devices and other critical environments. Finally, adding GO to LNBGSS improved the resultant composite properties to be used in different applications.

The biocompatibility of GO is a complex and sensitive aspect influenced by numerous factors related to both the material itself and the interacting organisms or cells. Given GO’s potential as a future-oriented platform, thorough evaluation and characterization of its interactions with biological systems are highly recommended. We plan to conduct more precise analyses to better understand individual physiological responses and establish toxicity standards.

## Data Availability

The data that support the findings of this study are available from the corresponding author upon reasonable request.
